# September and the Panel Doctor

**Published:** 1923-09

**Authors:** R. W. Harris


### SEPTEMBER AND THE PANEL DOCTOR.

By R. W. HARRIS.

THE month of September this year has for panel doctors a peculiar interest. Before the month has run its course the Minister of Health will, it is anticipated, have made up his mind as to the amount of the capitation fee which will be proposed as the rate of the panel doctor's remuneration after the end of the present year. Doctors are refraining from any public discussions on the subject until the amount of the Minister's "offer" is made known. They are showing this reticence on the advice of the Executive of the Conference of Panel Committees in order that the hands of that central body may not be fettered in their negotiations with the Government. In the course of a few days after the Minister has made his announcement, the Panel Committees up and down the country will have before them for immediate discussion the terms of that announcement, with the comments of their Executive, and they will meet together in conference on October 18th to decide upon their course of action.

### A Wrong Atmosphere.

We seem to be getting back once more into the atmosphere of struggle which appears inseparable from this question of the pay of the panel doctor. There is a suggestion abroad that the decks are being cleared for action. There is in existence, we know, a plan of campaign for use in what are darkly referred to as "certain eventualities." The word "strike," it is true, is deprecated in responsible medical quarters, but it is a facile expression which will be readily used in the Press, and therefore by the public, in the event of the doctors as a body refusing to work the National Health Insurance scheme as a public service, whatever their alternative scheme may be.

### Why Should there be a Fight?

The doctors will, of course, settle the question with the Government in their own way, but it seems rather a pity that so many of them think, or let it be inferred that they think, that a struggle is inevitable. I do not know what there is in their past experience of dealings with Ministers to lead them to suppose that the rate of pay will only be arrived at after the sort of higgling that is common to the market-place. Perhaps the danger of a flank attack by the Approved Societies has something to do with it.

### What Happened in 1911–12.

The Government in 1911-12 had provided in the financial scheme of the Insurance Act an amount for the payment of the doctors arrived at after extensive investigation of the conditions obtaining in club practice and by a considerable loading of the rates of payment which were found to obtain, because it was known that those in many cases were nothing better than sweated rates. Notwithstanding this, the Government found that they were wrong. They satisfied themselves that they would not get an adequate and reasonably contented service unless they increased the proposed capitation fee by half-a-crown. They obtained such a service, and the taxpayers found themselves with a new charge of about two millions a year.

### What has Happened Since?

In the subsequent history of the panel doctor's pay, I am unable to find any tendency on the part of the Government to higgle, that is, to propose a figure less than they are prepared to pay upon gentle, or possibly vigorous, pressure being applied. The course pursued by the capitation fee shows that in the closing days of the war the Government supplemented the capitation fee by amounts corresponding, as closely as was practicable, with the amounts paid to public servants as war bonus. With economic conditions changing, and the value of the sovereign depreciating, the Minister offered an increased capitation fee of eleven shillings. The matter went to arbitration, and the arbitrators awarded eleven shillings. Later, a payment of 9s. 6d. was said by the Government to be adequate to secure a reasonably contented service, and this proved to be the case, even though the contentment of the doctors was in part to be attributed to a consciousness of a cheerful sacrifice in the public interest. In all this, I say, I find no disposition on the part of the responsible Minister to higgle.

### What Will Happen Now?

I am one of those, possibly simple-minded, people who think that the Minister will again put forward for acceptance an amount which will secure for him an adequate, reasonably contented service. The doctors may as well make up their minds that some reduction of the present capitation fee is inevitable. Anyone who has taken the trouble to read the public utterances of the head of the present Government must be aware that in his view economic recovery must precede social reform, even when social reform is something more definite and tangible than that which would lie hidden in an extra sixpence or shilling to the panel doctors. But when I read in the *Times* that some "competent observers" are of opinion that a fee of 7s. or even less would be accepted, I take leave to doubt the competence of these observers. On the other hand, it is difficult to understand the object of the Panel Committees in passing a somewhat unreal resolution that anything less than 11s. is inadequate. Too little importance is attached in references to this question to the fact that the doctors are asking for a five years' settlement. It is earnestly to be hoped that a settlement for that period, at least, will be made. But it cannot be gainsaid that it introduces an element which defies precise evaluation, and would make arbitration a matter of difficulty. The Minister would probably find an answering echo in public opinion, which is impatient of complicated settlements, if he said :—"In 1912 my predecessor made the famous half-a-crown settlement for five years. Some very capable arbitrators fixed 11s. a few years ago. Conditions have changed very much since then, and the future is an unknown quantity. I will take half-a-crown off. That will leave 8s. 6d." There is something of stability and substance about the half-crown as a coin which communicates itself to "half-crown" settlements.

